# Effects of a nutritional supplement in dogs affected by osteoarthritis

**DOI:** 10.1002/vms3.182

**Published:** 2019-07-17

**Authors:** Nadia Musco, Giuseppe Vassalotti, Vincenzo Mastellone, Laura Cortese, Giorgia della Rocca, Maria Luce Molinari, Serena Calabrò, Raffaella Tudisco, Monica Isabella Cutrignelli, Pietro Lombardi

**Affiliations:** ^1^ Department of Veterinary Medicine and Animal Production University of Napoli Federico II Naples Italy; ^2^ DVM Vinchiaturo Campobasso Italy; ^3^ Department of Veterinary Medicine University of Perugia Perugia Italy; ^4^ Dynamopet srl Verona Italy

**Keywords:** chondroprotector, herbal medicine, lameness, nutraceuticals, osteoarthritis

## Abstract

Osteoarthritis is a form of chronic joint inflammation caused by the deterioration of the joint cartilage, accompanied by chronic pain, lameness and stiffness, particularly after prolonged activity. Alternative treatments of canine osteoarthritis would be desirable and, recently nutraceuticals, have been proposed for this purpose. Twenty cross breed adult dogs affected by osteoarthritis were enrolled and equally divided into two groups (control vs. experimental). The nutritional supplement (Dynamopet srl, Verone, Italy) was administered for 90 days to the dogs of the experimental group in order to evaluate its metabolic and locomotor effects. All the clinical signs (lameness, pain on manipulation and palpation, range of motion and joint swelling) significantly (*p* < 0.01) improved during the trial as regards the experimental group. This group showed a significantly lower joint score than the control group (mean value 7.40 vs. 3.80). With regard to haematology, the mean corpuscular volume resulted significantly (*p* < 0.01) higher in the experimental group, i.e. alkaline phosphatase, cholesterol and triglycerides values decreased and were significantly (*p* < 0.01) lower than the control one, thus suggesting an improvement in bone remodelling and lipid metabolism. A decrease in the reactive oxygen metabolites and an increase in the biological antioxidant potential demonstrated an improvement in oxidative stress during the trial in the experimental group compare to the control group. Interleukins 6 decreased in the experimental group, while interleukins 10 resulted in the opposite trend. Moreover, the administration of up to 3 months of the studied supplement was well tolerated in the dogs and caused no adverse effects.

## 1 INTRODUCTION

Osteoarthritis (OA) is a form of chronic joint inflammation caused by the deterioration of the joint cartilage. It is highly prevalent in dogs (Aragon, Hofmeister, & Budsberg, 2007; Paster et al., 2005; Smith et al., 2006), mainly in overweight and large breed dogs. Some breeds (i.e. the Labrador Retriever, the German Shephard etc.) are even genetically predisposed to develop arthritis (Anderson et al., 2018). There is no known cause for primary OA, while there are a wide variety of causes for secondary OA, such as traumas, abnormal wear on the joints and cartilages, the hip or elbow dysplasia, the dislocation of the kneecap or shoulder, and dissecans osteochondritis. Finally, obese dogs, because of the high stress levels on their joints, and dogs with disorders such as diabetes, prolonged steroid treatment, and hyper laxity may also be at a higher risk of OA (Sanderson, 2012). The disease is accompanied by chronic pain, lameness and stiffness, particularly after prolonged activity.Their quality of life is reduced, leading finally to the loss of joint functions and mobility. Currently no cure exists and the pharmacological treatment is limited to clinical signs alleviation. For this reason, the therapeutic management of OA in dogs is dominated by non‐steroidal anti‐inflammatory drugs (NSAIDs), which are only able to treat the symptoms of OA by decreasing the pain and inflammation (Curry, Cogar, & Cook, 2005; Innes, Fuller, Grover, Kelly, & Burn, 2003). Unfortunately, the use of NSAIDs may be associated with detrimental effects, especially gastrointestinal adverse effects (Buttgereit, Burmester, & Simon, 2001). Besides pain relief, preventing the cartilage degradation is an important objective for the treatment and requires the long‐term use of safe modalities. Indeed, the absence of any cure reinforces the importance of prevention (Innes et al., 2003). For these reasons, alternative treatments of canine osteoarthritis would be desirable and, recently, nutraceuticals have been proposed for this purpose (Colitti, Gaspardo, Della Pria, Scaini, & Stefanon, 2012; Henrotin, Kurz, & Aigner, 2005; Innes et al., 2003). In addition, some herbal medicinal products (nutraceuticals of plant origin) have been shown to interact with the mediators of the inflammation and, therefore, may be used in the treatment of OA (Cameron et al., 2009). These products can also act as free radical involving use through other mechanisms. However up to now, few clinical trials have been carried out to substantiate the safety and efficacy of herbal medicines, which are also not free of potentially serious adverse effects.

Glucosamine and chondroitine sulphate are popular supplements used to treat the pain and loss of functions associated with osteoarthritis. Several studies on dogs showed significant improvement in pain scores with the administration *per os* of both substances (D'Altilio et al., 2007; Gupta et al., 2012; Steagall et al., 2007; Vassalotti et al., 2017).

*Ribes nigrum* is a perennial shrub which is native of central Europe and northern Asia. The phenolic and anthocyanin compounds, in association with the high levels of vitamin C, confer on the blackcurrant fruit specific antioxidant capacities (Moyer, Hummer, Finn, Frei, & Wrolstad, 2002). Regarding the anti‐microbial effects, anthocyanins have been demonstrated to possess antimicrobial capabilities against a variety of bacteria, in particular Gram+ (Puupponen‐Pimia et al., 2001).

The Krill oil*,* extracted from the Antarctic krill (*Euphausia superba*), is known for its anti‐oxidative effect, due to astaxanthin, which keeps the ω‐3 polyunsaturated fatty acids (PUFA) intact and thereby preserves them from oxidation (Tou, Jaczynski, & Chen, 2007; Winther, Hoem, Berge, & Reubsaet, 2011). An increasing number of studies on animals as well as on humans demonstrate the health benefits of PUFA, in particular EPA (C20:5 ω‐3) and DHA (C22:6 ω‐3) (Dawczynski, Jentsch, Schweitzer, Hammer, & Strobel, 2013; Nestel et al., 2015). Cardiovascular benefits have been reported, providing an anti‐inflammatory effect (Sudheendran, Chang, & Deckelbaum, 2010).

Shiitake (*Lentinus edodes*) possess interesting nutritional characteristics and medicinal properties used by oriental cultures. Shiitake mushroom is known for several therapeutic actions such as antioxidant and antimicrobial properties, because of the diversity of its components (such as phenolic acids and tocopherols) which are able to trap reactive oxygen/nitrogen species and inflammatory mediators (Zembron‐Lacny, Gajewski, Naczk, & Siatkowski, 2013). In addition, Lentinula edodes‐derived nondigestible carbohydrates have been shown to possess health benefits on gut microbiota (Xu & Zhang, 2015).

*Equisetum arvense*, native to the Arctic and temperate regions of Europe, belongs to the *Equisetopsida* family, and it is commonly used as a herbal remedy. The phytotherapic properties are anti‐bacterial (Bessa Pereira et al., 2012; Milovanović, Radulović, Todorović, Stanković, & Stojanović, 2007), antioxidant (Cetojević‐Simin et al., 2010; Wu et al., 2010) and anti‐inflammatory (Gründemann et al., 2014).

*Equisetum arvense* is especially known for its mineralizing activity. In the bone tissue the silicon enables the deposit of calcium in the active sites of bone calcification, especially in the early stages of growth (Jugdaohsingh, 2007).

*Boswellia serrata* is a branching tree native to the hilly regions of India. It produces an aromatic resin that is used for medicinal purposes. Comblain, Serisier, Barthelemy, Balligand, and Henrotin (2015) showed that Boswellia resin administered for 6 weeks in dogs helped to reduce lameness (when moving or after a long rest), local pain and stiff gait (Reichling, Schmokel, Fitzi, Bucher, & Saller, 2004). The anti‐inflammatory effects of Boswellia and boswellic acids have been linked to the ability to inhibit the synthesis of leukotrienes, the chemical mediators of the inflammatory process in various inflammatory diseases, including arthritis (Kimmatkar, Thawani, Hingorani, & Khiyani, 2003).

Both *Boswellia serrata* and *Equisetum arvense* are mentioned in phytovigilance as possible modulators of cytochrome P450 action but the modulating action of these two medicinal plants seems strongly dose‐related. In 2006, Frank and Unger described *Boswellia serrata* as an inhibitor of numerous cytochrome P450 enzymes, while more recently, Dey, Chaskar, Athavale, and Chitre (2015) when using *Boswellia serrata* in association with other medicinal plants, did not find such inhibitory activity and hypothesized that a particularly high dose of Boswellia extract is required to act on cytochrome P450 enzymes.

A similar cytochrome inhibitory action was also described as regards *Equisetum arvense* (van den Bout‐van den Beukel, Koopmans, van der Ven, De Smet, & Burger, 2006), reporting on how the action of antiretroviral drugs is reduced by the simultaneous intake of the preparation based on some medicinal plants, including *Equisetum arvense*.

*Curcuma longa* is an aromatic substance of vegetable origin (spice) with numerous biological activities, anti‐inflammatory, antioxidant as well as cardiovascular benefits (Chattopadhyay, Biswas, Bandyopadhyay, & Banerjee, 2004; Teiten, Eifes, Dicato, & Diederich, 2010). These effects are due to diarylheptanoid compounds known as curcuminoids, which are considered the main active principles of the plant, although their bioavailability was shown to be poor because of scarce absorption, rapid metabolism and systemic elimination (Ireson et al., 2001). *Curcuma longa* has been widely studied for its modulating capacity of cytochrome P450 action. In particular, it has been shown that the plant possesses the ability to inhibit the activity of CYP2A1 and CYP2B1 (Walle & Walle, 2007). Nandal, Dhir, Kuhad, Sharma, and Chopra (2009) reported an anti‐inflammatory activity with regard to *Curcuma longa*, and a possible synergy with the most common non‐steroidal anti‐inflammatory drugs, and so enhancing their anti‐inflammatory action without increasing the incidence of harmful side effects.

*Harpagophytum procumbens*, also called Devil's claw, contains in its roots two main bioactive components, harpagoside and harpagide, implicated in its anti‐inflammatory effects. It is a herbal substance commonly found in southern African countries, where its tubers have been used by native people to treat rheumatism, arthritis, inflammation, and stomach disorders (Fiebich, Muñoz, Rose, Weiss, & Mc Gregor, 2012; Inaba, Murata, Naruto, & Matsuda, 2010; Sanders & Grundmann, 2001). The anti‐inflammatory activity of harpagide may alleviate bone‐associated diseases, in the symptomatic treatment of osteoarthritis (Brien, Lewith, & McGregor, 2006), arthritis and other rheumatic diseases (Setty & Sigal, 2005). Various studies excluded interaction between *Harpagophytum procumbens* root and cytochrome P450 (Modarai, Suter, Kortenkamp, & Heinrich, 2011). On the other hand, an in vitro test demonstrates its inhibitory activity in the expression of cyclooxygenase 2 (COX‐2), an enzyme involved in the inflammatory processes of osteoarthritis (Abdelouahab & Heard, 2008). Another in vitro studied evaluated the effect of the *Harpagophytum procumbens* on the inflammation induced by lipopolysaccharide on fibroblasts in mice, showing that its aqueous extract inhibits the synthesis of prostaglandin E2 and nitric oxide by inhibiting the increase in COX2 and the expression of mRNA specific for the inducible Nitric Oxide Synthase (iNOS) gene (Jang et al., 2003). By contrary, other authors reported that the administration of *Harpagophytum procumbens* did not affect plasma concentrations of prostaglandins and other eicosanoids, therefore hypothesizing a mechanism different from that of the common NSAIDs (Moussard, Alber, Toubin, Thevenon, & Henry, 1992). For this reason the clinical use of *Harpagophytum procumbens* is not complicated by the gastrointestinal side effects typical of NSAIDs and can be favorably associated with their use; even if it has been recommended that use should be not prolonged and/or interrupted by periods of suspension (Firenzuoli, 2001).

These results indicate that *Harpagophytum procumbens* possesses an anti‐inflammatory action which is expressed through the suppression of COX2 and the expression of iNOS.

Oxidative stress is implicated in the pathogenesis of many diseases and inflammatory conditions. Reactive oxygen species (ROS) are products of a normal cellular metabolism and are formed during the enzymatic reactions of intercellular and intracellular signalling. Physiological and/or pathological mechanisms can cause an over‐production of ROS and, consequently, oxidative stress, i.e. a state in which an oxidant‐generating system overcomes an antioxidant defence system. The process of oxidative phosphorylation might be affected because of a possible damage to proteins and membrane lipids (Panda, Patra, Nandi, & Swarup, 2009). It is known that the association of different compounds in phytotherapy often exerts a synergic activity. If this is so, the association of these components should guarantee a beneficial effect by acting on the different pathways of the oxidative stress and inflammatory response (Mastellone et al., 2016).

Although some information is available in the literature regarding the substances included in the formula, no study has tested the effectiveness of their combination. Indeed, no information concerning metabolic effects, tolerance and benefits is presently available for this nutritional supplement. Further, the use of nutraceuticals as well as phytotherapics in veterinary medicine is not often supported by an exhaustive scientific literature.

The aim of the present study was to verify the effects of the administration of Dì*namic* ^™^ (Dynamopet srl, Verone, Italy) in dogs affected by OA.

## 2 METHODS

### 2.1 Ethic statement

This study has been reviewed by the Ethical Animal Care and Use Committee of the University on Napoli Federico II and received formal Institutional approval (prot. N 71185/2015 on the 21st of July 2015) in accordance with local and national law, regulations and guidelines. This study avoided any discomfort for the animals by using proper clinical management. The blood collection was carried out after infurming the owner and getting their consent as based on the national guidelines for animal welfare (no cruelty, providing no segregation, even partial, of the animals).

### 2.2 Supplement

The commercial supplement Dì*namic* ^™^ (Dynamopet srl, Verone, Italy), is an association of: Glucosamine sulphate 10%, Krill oil 3%*,* Chondroitine sulphate 1.25%, *Ribes nigrum*, Krill flour 1%, *Lentinus edodes*,* Equisetum arvense,* and products obtained from the transformation of herbs (*Curcuma longa*,* Boswellia serrata*,* Harpagophytum procumbens*). It aims to improve both acute and chronic arthritis in dogs and to increase performance in athlete dogs.

### 2.3 Animals and study design

Twenty cross breed dogs affected by OA (12 males and 8 females; mean age 9.2 ± 5.1 years old) were enrolled with the owners' consent. The study was performed on household dogs to avoid any possible interference dependent on the usual environmental changes. All the dogs were fed for 4 months (including 1 month of adaptation) on a commercial diet (crude protein 211 g kg^−1^, acid hydrolysed ether extract 90 g kg^−1^, crude fiber 39 g kg^−1^, 3110 kcal kg^−1^ as a feeding basis) administered using the ratio of 130 kcal ME per kg^0.75^ of ideal body weight. All the dogs were enrolled with a clinical diagnosis of OA also based on X‐rays revealing the beginnings of cracks in the cartilage and the growth of bone spurs. The dogs showed, at least, intermittent mild lameness and pain in the arthritic joint level. No dogs showing comorbidities were included in the trial.

After enrollment, the dogs were randomly equally divided into two groups (control group and experimental group), homogeneous by sex (6 males and 4 females each). The animals received no pharmacological treatment for at least 2 months before and all through the trial.

The study was performed in double blind. Dì*namic* ^™^ (Dynamopet srl, Verone, Italy) was orally administered to the dogs of the experimental group using the ratio of 0.5 ml kg^−1^ of live weight for 90 consecutive days, once a day approximately at 9:00 p.m. and placebo using the ratio of 0.5 ml kg^−1^ of live weight was administered to the dogs of the control group in order to minimize any false results.

### 2.4 Clinical evaluation

The health status of all the dogs was assessed through blood analyses and a clinical examination. These practices were carried out by the same vet, before starting the treatment (0) and for each 30‐day period up to 90 days (30, 60 and 90). Physical examinations, including lameness evaluations (overall lameness, pain on manipulation and palpation, joint swelling and range of motion), were done at all the aformentioned time points as proposed by Pollmeier, Toulemonde, Fleishman, and Hanson (2006). The scores for lameness, pain on manipulation and palpation, range of motion and joint swelling were assigned to the most severely affected limb, as determined at the initial evaluation. Overall pain during limb manipulation was evaluated by the animals' vocalization or other psychomotor changes or pain expressions during the extension and flexion of all four limbs for few min. For each variable, results were graded on a scale of 0–3: 0 (none), 1 (slight), 2 (moderate) 3 (severe) and, an overall score was calculated while placing emphasis on the Pollmeier et al. (2006) lameness evaluation:

$$\begin{array}{cc} & \left(2\times \text{lameness})+(\text{pain on manipulation/palpation})+(\text{range of motion}\right)\\ & \;+(\text{joint swelling}) \end{array}$$

### 2.5 Blood sampling and analyses

Blood samples were collected monthly from the fasting dogs of both groups (at 0, 30, 60 and 90 days) from the jugular vein into tubes with and without K3‐EDTA, and immediately transported to the laboratory. Serum was obtained by centrifugation at 1200*g* for 15 min, divided into aliquots and frozen at −80°C.

A complete cell blood count was performed for each whole blood sample within 30 min of the collection by a Lasercyte^™^ haematology analyser (IDEXX Laboratories Inc., Westbrook, Main, USA) that provides complete blood counts: white blood cells (WBC), red blood cells (RBC), hemoblobin (HGB), hematrocrit (HCT), mean corpuscular volume (MCV), mean corpuscular hemoglobin concentration (MCHC), platelet (PLT), mean platelet volume (MPV), blood cell distribution width (RDW), lymphocytes (LYM), monocytes (MON) and granulocytes (GRA).

Blood chemistry analyses on serum aliquots were performed by an automatic biochemical analyser (AMS Autolab, Diamond Diagnostics, USA) using reagents from Spinreact (Girona, Spain) to determine: total proteins (TP), albumin (ALB), creatinine (CREA), glucose (GLU), aspartate amino transferase (AST), alanineaminotransferase (ALT), gamma‐glutamyltransferase (GGT), bilirubin (BIL), lactic dehydrogenase (LDH), alkaline phosphatase (ALP), creatine phosphokinase (CPK), cholesterol (CHOL) and triglycerides (TRI).

Reactive oxygen metabolites (the d‐ROMs test evaluates the free alcohoxyl and hydroperoxyl radicals derived from hydroperoxides present in the sample), oxy‐adsorbent (the OXY test evaluates the total antioxidant barrier understood as the whole of the chemically active capacity) and biological antioxidant potential (the BAP test evaluates so as to allowthe chemically active antioxidant capacity of the plasma barrier to be measured) were also performed on serum aliquots using reagents from Diacron International s.r.l. (Grosseto, Italy) validated for canine species (Pasquini et al., 2008).

Canine Interleukins 6 (IL‐6) and 10 (IL‐10) and Canine Tumour Necrosis alpha (TNF‐α) were detected in serum using Elisa kits (Genorise, Philadelphia, USA).

### 2.6 Statistical analysis

The blood count, blood biochemistry and lameness evaluation data were compared by one‐way ANOVA (JMP software, SAS Institute, NC, USA) according to the following model:

yijk=μ+Gi+Tj+G×Tij+1ijk.

where: *y* is the dependent variable, *μ* is the mean, *G* is the group effect (*i* = Control, Experimental), *T* is the time effect (*j* = 0, 30, 60, 90), *G* × *T* is the first level of interaction and ε is the error effect.

When significant differences were found in the ANOVA, means were compared using Tukey's test.

## 3 RESULTS

### 3.1 Clinical evaluation

All the clinical signs (lameness, pain on manipulation and palpation, range of motion and joint swelling) significantly (*p* < 0.01) improved along the trial in the experimental group, which showed significant lower joint score than the control group (mean value 3.80 vs. 7.40). Regarding the time effect, the improvement on the overall experimental group score resulted significantly (*p* < 0.01) lower at the two last samplings (mean value 7.70 vs. 2.30 at 60 days and 8.40 vs. 1.30 at 90 days for the experimental group and the control group, respectively). The interaction between groups resulted as being highly significant (*p* < 0.0001) (Table 1).

**Table 1:** Clinical evaluation of control and experimental groups at days 0, 30, 60, 90 in OA dogs

Group effect	C	E
	7.40A	3.80B
Time effect	C	E
0	6.60AB	6.40AB
30	6.90AB	5.00BC
60	7.70B	2.30CD
90	8.40A	1.30D
G × T	<0.0001	
RMSE	2.022	

Note: Within a column, means without a common letter differ (capital letters: *p* < 0.01 A–D).

Abbreviations: C, Control group; E, Experimental group; RMSE, Root‐mean‐square error.

### 3.2 Haematology and blood chemistry

All the parameters fall within the normal range for adult dogs and no signs of adverse effects were observed after the clinical examination, showing that supplementation was well tolerated. Despite that, some significant differences were detected.

Concerning haematology (Table 2), MCV resulted as being significantly (*p* < 0.01) higher in the experimental group compared to the control one. MCHC resulted as being significantly (*p* < 0.05) lower during the final sampling (time 90) but no differences were observed between the groups.

**Table 2:** Haematology of the control and experimental groups at days 0, 30, 60, 90 in OA dogs

	RBC	HTC	HGB	MCV	MCH	MCHC	RDW	RETIC	WBC	NEU	LYM	MONO	EOS	BASO	PLT	MPV	RDW	PCT
Group effect	M μl^−1^	%	g dl^−1^	fL	pg	g dl^−1^	%	K L^−1^	K μl^−1^	K μL^−1^	K μL^−1^	K μL^−1^	K μL^−1^	K μL^−1^	K μL^−1^	fL	%	%
C	6.92	50.75	16.74	67.61B	22.28	32.55	15.38	59.10	8.57	5.93	1.59	0.730	0.340	0.045	280.15	10.32	21.67	0.312
E	7.17	50.99	16.57	71.72A	22.57	32.46	15.32	62.86	8.79	6.00	1.65	0.742	0.320	0.028	275.55	10.83	21.62	0.320
Time effect																		
0	6.70	48.88	17.38	68.03	22.23	34.33a	14.95	56.93	8.46	6.10	1.25	0.745	0.305	0.035	269.95	10.77	21.30	0.270
30	6.97	50.48	16.34	70.83	21.81	32.21ab	15.26	61.93	8.53	5.80	1.65	0.670	0.325	0.035	285.80	10.42	22.59	0.340
60	7.26	52.22	16.19	69.44	22.40	32.77ab	15.29	68.61	9.04	6.17	1.72	0.775	0.325	0.045	283.15	10.01	21.85	0.330
90	7.23	51.92	16.70	70.35	23.17	30.72b	15.90	56.45	8.70	5.79	1.84	0.755	0.365	0.030	277.65	11.10	20.84	0.325
*G* × *T*																		
*p*	0.991	0.088	0.176	0.247	0.481	0.222	0.274	0.882	0.790	0.800	0.902	0.926	0.958	0.916	0.103	0.980	0.862	0.821
RMSE	0.798	4.506	1.624	5.079	3.556	0.091	1.909	15.86	1.505	1.440	0.472	0.255	0.140	0.052	41.970	2.037	2.077	0.089

Note: Within a column, means without a common letter differ (capital letters: *p* < 0.01 A–B; lower case letters: *p* < 0.05 a–b).

Abbreviations: C, Control group; E, Experimental group; RMSE: Root‐mean‐square error.

Concerning blood chemistry (Table 3), the ALP resulted as being significantly (*p* < 0.01) lower in the experimental group compared to the control one (113.10 vs. 153.72 U L^−1^, respectively). CHOL significantly decreases (*p* < 0.01) in the experimental group (189.45 vs. 207.70 mg dl^−1^, for E and C groups, respectively) as well as triglycerides (71.47 vs. 96.94 mg dl^−1^ for E and C groups, respectively).

**Table 3:** Blood chemistry parameters of the control and experimental groups at days 0, 30, 60, 90 in OA dogs

	AST	ALT	GGT	ALP	BIL	GLU	PT	ALB	UREA	CREA	LDH	CPK	CHOL	TRI
Group effect	U L^−1^	U L^−1^	U L^−1^	U L^−1^	mg dl^−1^	mg dl^−1^	g dl^−1^	mg dl^−1^	mg dl^−1^	mg dl^−1^	U L^−1^	U L^−1^	mg dl^−1^	mg dl^−1^
C	48.35	26.34	3.93	153.72A	0.757	86.10	7.00	3.95	22.69	0.982	140.25	36.42	207.70A	96.94A
E	46.57	26.90	3.98	113.10B	0.725	87.77	7.06	3.83	22.34	0.962	157.55	37.48	189.45B	71.47B
Time effect														
0	48.45	24.30	3.92	156.05A	0.785	86.20	7.96	3.85	22.65	0.970	119.00	35.28	214.60A	107.10A
30	48.15	25.40	3.82	148.45A	0.760	89.05	7.10	4.01	21.85	0.910	164.55	36.80	206.60A	84.95AB
60	49.20	26.72	3.92	129.70A	0.755	83.65	7.05	3.92	22.05	1.040	153.70	39.62	198.30AB	75.47B
90	44.05	30.05	4.15	99.45B	0.665	88.85	7.01	3.79	23.50	0.970	158.35	36.11	174.80B	96.30B
*G* × *T*														
*p*	0.920	0.889	0.600	0.012	0.260	0.599	0.336	0.586	0.935	0.644	0.395	0.991	0.921	0.162
RMSE	11.090	7.630	0.863	34.530	0.234	7.425	0.487	0.276	4.419	0.280	56.590	9.580	33.290	32.460

Note: Within a column, means without a common letter differ (capital letters: *p* < 0.01; lower case letters: *p* < 0.05).

Abbreviations: C, Control group; E, Experimental group; RMSE, Root‐mean‐square error.

Concerning the time effect the ALP resulted as being significantly (*p* < 0.01) lower at the end of the experimental period (156.05, 148.45, 129.70 and 99.45 for 0, 30, 60 and 90 days, respectively). The cholesterol resulted as being lower at the final sampling (214.60, 206.60, 198.30 and 174.80, at 0, 30 60, 90 days, *p* < 0.01, respectively). The TRI significantly (*p* < 0.01) decreases from the 60‐days period until the end of the experimental period (107.10, 84.95, 75.47 and 96.30 for 0, 30 60, 90 days, respectively).

The interaction between the groups and the time effect resulted as being significantly only for the ALP parameter (*p* < 0.012).

Regarding the oxidative status (Table 4) the d‐ROMs significantly decreased (*p* < 0.01) in the experimental group as compared to the control one (55.06 vs. 65.51 U CARR, respectively). Similarly, an improvement in the biological antioxidant potential, as confirmed by the significant (*p* < 0.05) increase in the BAP levels, was detected.

**Table 4:** The Oxidative and inflammation status of the control and experimental groups at days 0, 30, 60, 90 in OA dogs

	d‐ROMs	BAP	OXY	TNF‐α	IL‐6	IL‐10
Group effect	U CARR	μmoli/L	mmoli/L	pg/ml	pg/ml	pg/ml
C	65.51A	4232.80b	193.47	134.62	206.52A	114.09B
E	55.06B	4506.70a	196.50	121.18	178.82B	132.38A
Time effect						
0	74.65A	4171.15b	188.35	123.73	211.20A	106.88B
30	64.68B	4133.10b	191.50	139.40	235.55A	124.65AB
60	53.53C	4480.00ab	203.70	133.25	157.25B	119.41AB
90	48.30C	4694.75a	196.40	115.23	166.68B	141.99A
*G × T*						
*p*	0.195	0.139	0.516	0.962	0.001	0.199
RMSE	10.95	451.86	28.46	27.93	41.73	30.11

Note: Within a column, means without a common letter differ (capital letters: *p* < 0.01; lower case letters: *p* < 0.05).

Abbreviations: C, Control group; E, Experimental group; RMSE, Root‐mean‐square error.

No significant differences were recorded for the OXY parameter.

The IL‐6 significantly (*p* < 0.01) decreased in the E group (206.52 vs. 178.82 pg ml^−1^ for the control and experimental group, respectively). On the contrary, the IL‐10 significantly (*p* < 0.01) increased (114.09 vs. 132.38 pg ml^−1^, respectively). No differences were observed for the TNF**‐**α.

Regarding the time effect d‐ROMs significantly (*p* < 0.01) decreased from 30 days up to 90 days. On the contrary, the BAP significantly (*p* < 0.05) improved at the end of the trial. The IL‐6 resulted as being significantly (*p* < 0.01) lower at both 60 and 90 days. The IL‐10 resulted higher (*p* < 0.01) at 90 days.

The interaction between the groups and the time was significant (*p* < 0.01) only for IL‐6.

## 4 DISCUSSION

Osteoarthritis is one of the most common causes of lameness in dogs. It is caused by a deterioration of the joint cartilage followed by pain, inflammation and loss of range of motion of the joint. Pharmacological treatment of OA is usually limited to NSAIDs, but their use is associated with potential side effects, which can be quite adverse (Aghazadeh‐Habashi & Jamali, 2011). Therefore, nutritional supplements improving the state of patients affected by osteoarthritis when they are tolerated well can be of great usefulness for the management of OA dogs.

### 4.1 Clinical examination

The improvement of the overall score in the experimental group was principally ascribable to the reduction of the pain of manipulation (the incidence of subjects with a severe/moderate degree of pain decrease from 50 to 0% from the first to the third observation). Also, the other clinical parameters of the Pollmeier scale showed a significant (*p* < 0.05) improvement during the trial. These results are probably due to the association of different active compounds in the formulation of the nutritional supplement. Indeed, the possible synergic activity among the various compounds should be assessed by specific studies. The anti‐inflammatory activity of the Devil's claw may be critical in the symptomatic treatment of osteoarthritis (Brien et al., 2006) but, also, glucosamine and chondroitin are nutritional supplements which have recently gained widespread use as per *os* treatment options for OA. There is evidence supporting that both these compounds reach and retain a certain concentration in plasma and in joint fluid and cartilage (Innes, Clayton, & Lascelles, 2010; Persiani et al., 2007; Vasiliadis & Tsikopoulos, 2017). Moreover, also *Boswellia serrata*, mainly because of its boswellic acids, is already known as a mediator in various inflammatory diseases. As reported by Kimmatkar et al. (2003), the administration of *Boswellia serrata* extracts showed significant reduction in swelling and pain and an improvement in the motor skills in patients affected by osteoarthritis.

### 4.2 Haematology and blood chemistry

Clinical biochemistry as well as haematology showed that the supplementation was well tolerated. The absence of adverse effects is often a critical point in the management of chronic diseases. The differences in MCV values between those groups associated with a normal HCT have no relevant clinical sign. It is well‐known that the MCV decreases in chronic diseases (Thrall, Weiser, & Jain, 2004), even if all the values were comprised in the physiologic range for dogs.

In general, triglycerides in serum reflects inflammatory conditions, since they provide additional nutrients that help the metabolic requirements of the cells implicated in tissue repair processes (Popa, Netea, van Riel, van der Meer, & Stalenhoef, 2007). The decrease of the TRI registered in the E group and during the trial suggests a reduction in the inflammatory state in the dogs during the experimental period. Nowadays OA is not considered as only a joint disorder associated with mechanical stress and aging but also a ‘metabolic syndrome’ in which several risk factors work together contributing to disease initiation and/or development. High cholesterol levels have been reported as being one such metabolic risk factor. Farnaghi, Crawford, Xiao, and Prasadam (2017) reported that synovial fluid contains a low concentration of cholesterol compared to plasma levels whereas the synovial fluid of patients with osteoarthritis has a higher amount of cholesterol and cholesterocrystals than the synovial fluid of healthy individuals.

Abdurhman (2003) also reported an association between serum cholesterol and OA. Osteoarthritis can cause a reduced physical activity and thereby increase of triglycerides and cholesterol. On the other hand, an increase in cholesterol can be a predisposing factor to osteoarthritis.

The observed reduction in the TRI in the E group and from 60 days up to the end of the experimental period suggests an improvement of bone status and lipid metabolism and is probably due to the presence of Krill oil in the formula of the nutritional supplement (Berge et al., 2015). Interestingly enough, despite some of the compounds of the formula, this (using the ratio of 832.5 mg EPA and DHA per day), is reported to increase cholesterol levels (Berge et al., 2015), whereas a decrease in CHOL was detected in this trial for the experimental group as compared to the control one. Indeed, this may be due to the activity of the *Boswellia serrata* that possesses ipo‐cholesterolemic activity in humans (Ahangarpour, Heidari, Mard, Hashemitabar, & Khodadadie, 2014). This suggests that a studied association of different compounds might lead to beneficial effects. In this case, two opposite effects resulted in an overall improvement in the CHOL in the dogs.

### 4.3 Oxidative and inflammation status

*Equisetum arvense* and *Lentinus edodes* are probably responsible for the improvement of BAP associated with the decrease in d‐ROMs in the experimental group as compared to C. Experimental outcomes reported by Li et al. (2016) showed that *Equisetum* spp. is considered an antioxidant ingredient due to its ability to reduce free radical chain reactions. Moreover, *Lentinus edodes*, due to its phenolic compounds, possess antioxidant capacities, both in vitro (Mishra et al., 2013; Naknaen, Itthisoponkul, & Charoenthaikij, 2015) and in vivo (Reis, Martins, Barros, & Ferreira, 2012). Interestingly enough, OXY results were not statistically significant and such results should merit further studies. Both the BAP and OXY allow us to measure the chemically active antioxidant capacity (scavengers) but OXY analyzes the plasmatic structural components such as mucopolysaccharides, amino acids and proteins (shock absorbers) whereas the BAP includes antioxidants both of an exogenous nature (ascorbic acid and tocopherols) and of an endogenous nature (uric acid, bilirubin and albumin) (Lombardi et al., 2017; Morittu et al., 2018). Moreover, Vassalle et al. (2012) reported that the relationship between the global index and the oxidative markers is essentially driven by the dROMs test. The relative importance of pro‐oxidant and antioxidant counterparts on the estimation of the overall oxidative stress status can vary in each clinical or experimental setting and depends on the evaluated biomarker.

The significant decrease in the IL‐6 and the increase in the IL‐10 observed in the experimental group as compared to the control one and during the trial also confirm the beneficial effects of the nutritional supplement. The cytokines are the most universal regulatory system in inflammation and host defence against bacterial infection (Aggarwal, 2007; Popa et al., 2007). They can participate in immunopathologic process formation and function as diagnostic markers in some diseases.

*Harpagophytum procumbens* has been used for centuries to treat inflammatory diseases and recently, in Europe, it has been recommended for the management of pain and inflammation in OA (Haseeb, Ansari, & Haqqi, 2017). Clinical trials report the good tolerability and anti‐inflammatory effects of *Harpagophytum procumbens*. This plant exerts its anti‐inflammatory effects by suppressing the expression of many cytokines (Hostanska, Melzer, Rostock, Suter, & Saller, 2014; Inaba et al., 2010), in particular, an inhibition of IL‐1β, IL‐6, and TNF‐α in mice has been reported. In addition, as reported by many authors (Moon et al., 2006; Wang et al., 2009) curcumin also affects other molecular events implicated in the inflammation and the consequent promotion of tumor such as inflammatory cytokines (TNFα, interleukins IL‐1, IL‐6 and IL‐8).

Studies in vitro reported that *Ribes nigrum* is rich in α‐linolenic acid and possesses anti‐inflammatory activities, inhibiting the IL‐6 and TNF‐α expression, but it also has antioxidant capacities (Wang et al., 2009). Barre (2001) showed the in vivo anti‐inflammatory properties of blackcurrant oil, resulting in health effects after human consumption.

Moreover, Krill oil is rich in fatty acids implicated in the regulation of lipid metabolism, adipocyte differentiation and in inflammatory responses. Fish oil had been shown to alter pro‐inflammatory cytokine production (Zhu, Shi, Qian, Cai, & Li, 2008).

## 5 CONCLUSION

The results of this study support the evidence for the benefits of the nutritional supplement on animal welfare, improving joints and reducing pains in dogs with advanced osteoarthritis. The beneficial effects suggest that the different components included in the supplement may ameliorate the inflammatory state and oxidative stress in terms of cellular health by acting with different action mechanisms. Further studies are needed to assess the possible synergy among the different compounds in determining its overall effectiveness. Moreover, the administration which lasted up to 3 months of the studied supplement was well tolerated by the dogs who showed no adverse effects.

## CONFLICTS OF INTEREST

All authors declare no conflict of interest.

## SOURCE OF FUNDING

This research did not receive any specific funding. The nutritional supplement was provided by Dynamopet srl(Verone, Italy).

## ETHICAL STATEMENT

The authors confirm that the ethical policies of the journal, as noted on the journal's author guidelines page, have been adhered to and the appropriate ethical review committee approval has been received. The Directive 2010/63/EU was followed.

## AUTHOR CONTRIBUTIONS

Pietro Lombardi conceived and designed the experiments. Nadia Musco, Giuseppe Vassalotti, Raffaella Tudisco and Vincenzo Mastellone performed the experiments. Serena Calabrò and Nadia Musco analysed the data. Pietro Lombardi and Monica Isabella Cutrignelli contributed reagents/materials/analysis tools. Pietro Lombardi wrote the paper. Raffaella Tudisco, Maria Luce Molinari, Laura Cortese and Giorgia della Rocca, supervised the manuscript for intellectual content.
